# Evaluation of methods for detection of asymptomatic individuals infected with *Leishmania infantum* in the state of Piauí, Brazil

**DOI:** 10.1371/journal.pntd.0007493

**Published:** 2019-07-01

**Authors:** Gabriane Nascimento Porcino, Kátia Silene Sousa Carvalho, Débora Cavalcante Braz, Vladimir Costa Silva, Carlos Henrique Nery Costa, Isabel Kinney Ferreira de Miranda Santos

**Affiliations:** 1 Department of Biochemistry and Immunology, Ribeirão Preto School of Medicine, University of São Paulo, Ribeirão Preto, SP, Brazil; 2 Institute of Tropical Diseases Natan Portela, Federal University of Piauí, Brazil; Pasteur Institute of Iran, ISLAMIC REPUBLIC OF IRAN

## Abstract

**Background:**

Visceral Leishmaniasis in humans presents with fever, anemia, and splenomegaly and can be lethal if not treated. Nevertheless, the majority of *Leishmania infantum*-infected individuals does not manifest symptoms and remain so provided they are not immunosuppressed. In this work, the performance of different tests was evaluated to detect asymptomatic individuals who were living in Teresina, Piauí state, Brazil, an endemic area for VL.

**Methodology:**

*L*. *infantum*-specific antibodies were detected by ELISA and two different rapid immunochromatographic (IC) diagnostic tests, Kalazar Detect and OnSite, and parasitic loads were detected by real time PCR [qPCR]. Additionally, we measured levels of the biomarkers monokine induced by IFN-γ (MIG) and IFN-γ-induced protein 10 (IP-10) before and after stimulation of whole blood with soluble *Leishmania* antigen [SLA].

**Principal findings:**

Kalazar Detect and OnSite detected, respectively, 76% and 64% of patients presenting with active Visceral Leishmaniasis; 50% and 57% of patients remained positive in these tests, respectively, after treatment. Of the healthy participants in the study who were living in the endemic area, only 1.7% were positive with both of the IC tests. On the other hand, reactivity in ELISA tests revealed that 13% of these individuals presented asymptomatic infections; among VL patients, 84% presenting with active disease were reactive in ELISA, and after treatment, 55.5% were seropositive. *L*. *infantum* DNA was present in the blood of 37.9% of infected individuals living in the endemic area, while IP-10 and MIG biomarkers were detected in 26.7% of them. The greatest concordance of positivity occurred between ELISA and qPCR.

**Conclusion:**

The association of different techniques can detect asymptomatic infections, however, more research is necessary to develop ideal biomarkers that are simple to use in the clinic and in field studies in areas endemic for Visceral Leishmaniasis.

## Author summary

Infections with *Leishmania infantum* occurring in humans can become clinically manifest as active visceral leishmaniasis [VL], more frequently so in individuals with compromised immune systems and the disease can be fatal if left untreated. However, many individuals living in endemic areas who are infected through of a bite of the insect vector remain asymptomatic. Currently, other forms of transmission are being discussed, such as through blood transfusions and organ donations from asymptomatic individuals. Parallel to this knowledge, we know that dogs are reservoirs for *L*. *infantum*, but the role of asymptomatic humans as alternative reservoirs of VL is still is debated. A bone marrow aspirate is the best test to diagnose active VL in humans, but is too invasive to use in all people living in endemic areas. Therefore, less invasive tests are needed that use blood samples to detect infected persons who have not developed disease. In this regard, we evaluated different tests in order to find out which is the most effective to diagnose asymptomatic infections.

## Introduction

Visceral Leishmaniasis (VL) is classified by the World Health Organization as a neglected tropical disease due to high mortality rates, low attention given by the public sector and high endemicity in poor regions around world [1]. At a global level, ninety percent of VL cases were reported in seven countries, which include Brazil, Ethiopia, India, Kenya, Somalia, South Sudan and Sudan [1,2].

Caused by *Leishmania infantum* [synonymous *L*. *chagasi*] in the New World or *L*. *donovani* in the Old World, VL can be classified as an anthroponotic or zoonotic disease because it is transmitted between humans and others mammals, such as dogs [2,3]. Furthermore, blood transfusion is known to be another form of transmission of the parasite described in endemic areas and a cause for much concern since healthy uninfected donors are similar to asymptomatic infected donors. [4–6]. Indeed, in endemic areas in Brazil most people infected with *L*. *infantum* remain asymptomatic provided they are not immunosuppressed. Dogs are the main reservoirs of the disease, being well documented that asymptomatic canine hosts are frequent [7,8]. However, in addition to dogs, asymptomatic humans can represent an important reservoir and contribute to the maintenance of the pathogen in the endemic area [9,10].

The accurate epidemiological characterization of humans infected with *L*. *infantum*, as well as the elucidation of factors that result in asymptomatic infections or in development of active disease (VL) is essential for monitoring endemic areas and controlling this disease, thus the relevance of identifying asymptomatic individuals. This knowledge may contribute to development of control strategies and even treatments [11]. In this context, the clinical characteristics of active VL are shared with other diseases such as typhoid fever, tuberculosis and malaria, and coinfections of all types may occur, which make the diagnosis of VL complex [12].

Direct and indirect methods are used for diagnosis of VL. Direct methods consist of detection of the parasite through direct examination, culture or PCR using samples of tissue or marrow bone aspirates. The indirect methods screen for parasite-specific antibodies and for cellular responses with the leishmanin skin test (LST); antibody–based assays are immunofluorescence (IFAT), direct agglutination test (DAT), ELISA, Western blot and an immunochromatographic (IC) test (rk39) [13].

The first test capable of detecting infections in individuals without symptoms of the disease was the Montenegro LST used by Manson-Bahr, 1959 [14] but the reactant for performing it is currently not marketed in Brazil, thus requiring an alternative test for detecting asymptomatic individuals in an endemic area. A gold standard, a reliable method to detect individuals presenting with asymptomatic infections with causative agents of VL is still lacking, therefore it is difficult to determine the extent of infection rates. In addition, asymptomatic individuals alternately exhibit positive and negative reactions in different immunologically-based tests, thus making a negative test result difficult to interpret [8,15].

In summary, the identification, management and understanding of the biological and epidemiological significance of all categories of infected individuals has become a major challenge in programs for controlling VL as there are no validated markers yet, and the currently employed parasitological, molecular, and serological tools are not fully adequate. There is, therefore, an urgent need for new biomarkers that can identify the asymptomatic population in areas where viscerotropic species of *Leishmania* are endemic and which might also better assist in monitoring the success of treatment in patients with the active disease [16,17]. This work aims to evaluate different antibody-based diagnostic tests and the use of biomarkers produced by antigen stimulated cells, such as IFN-γ-induced protein 10 (IP-10, or CXCL-10) and monokine induced by gamma interferon (MIG, or CXCL9), for the detection of *L*. *infantum*-infected individuals without clinical manifestations of active VL in the city of Teresina, state of Piauí, Brazil, an endemic area for VL.

## Methods

### Volunteers

The participants of this study consist of patients with Visceral Leishmaniasis, before and after treatment, who were hospitalized at the Institute of Tropical Diseases Natan Portela in the city of Teresina, Piauí, Brazil, and volunteers without any symptom of the disease residing in the same city. Sample collections were carried out between August and November, 2017. Samples obtained from healthy volunteers residing in the city of Ribeirão Preto, São Paulo, Brazil, were used as control of non-endemic area.

The diagnosis of VL was confirmed by the positivity of amastigote forms in samples of bone marrow aspirate stained by Giemsa, and confirmed by cell culture in NNN medium.

### Ethics committee

The project was approved by the Research Ethics Committee by the Hospital das Clínicas of the Ribeirão Preto School of Medicine of the University of São Paulo, with Ethics Presentation Certificate number 67213017.0.0000.5440 and opinion number 2.101.755. The certificate for authorization for research at the Institute of Tropical Diseases Natan Portela is number AA.901.1.009518/17-65. All methods were performed according to the approved guidelines. A consent term was obtained from all study participants.

### Immunochromatographic tests

The assays were performed using Kalazar Detect Rapid (InBIOS International, Seattle, WA), according to the manufacturers' instructions: 20 μL of the sera were applied in the sample pad area from the test strip plus 3 drops of Chase Buffer, the results were read 10 minutes later and, in the positive samples, a control line and test line appear in the test area. In the OnSite Leishmania IgG/IgM Combo test (CTK Biotech), 20 μL of the sera were applied to the test area followed by 2 drops of diluents; in the positive samples a control line plus “G” and/or “M” lines appear, corresponding to IgG and IgM, respectively, according to the manufacturers' instructions.

### Preparation of soluble antigen of *L*. *infantum* (SLA)

Promastigote forms of *L*. *infantum* parasites (strain MHOM / BR / 74 / PP75) were cultured in Schneider's medium supplemented with 2% urine, 10% fetal bovine serum, 2% L-glutamine, 100 U / ml penicillin and 100 μg/mL streptomycin. The parasites in the stationary phase were enriched on the basis of negative agglutination by peanut agglutinin, and the soluble *Leishmania* antigen (SLA) was extracted. The promastigotes were washed with phosphate buffered saline (1X PBS) at 3,000 x g, 10 minutes, 4°C three times, re-suspended in Tris-HCl (pH 7.5) supplemented with protease inhibitors and subjected to 5 cycles of immersion alternately in liquid nitrogen and heated water baths. The lysate was sonicated, homogenized and centrifuged at 14,000 x g for 5 minutes at 4°C.

### ELISA

The 96-well plates were incubated overnight with 2 μg/ml SLA per well. The next day, they were washed using Tris plus tween 20 (TBT) buffer and blocked with 2.5% Molico milk plus tween 20 for 2 hours. The serum diluted 1:50 to 1:400 was added to the same blocking solution and incubated for 1 hour at 37°C. After washing, peroxidase conjugated protein G diluted at 1:15,000 in TBT buffer was added for 1 hour at 37°C. A further washing step was performed and the TMB substrate was added, the reaction was stopped with 0.2 N sulfuric acid and read at 450 nm in Multiskan GO, Thermo Scientific ELISA reader.

For each sample, the reactivity index (RI) was calculated by dividing the optical density of the serum test mean by the cutoff value which was determined by the mean optical density obtained by 16 negative samples plus three times the standard deviation. Samples were considered positive if RI ≥1.1 and negative if IR<1.1 as described in Marques et al. 2017 [11].

### DNA extraction from human blood

DNA extraction was performed using 200 μl of total peripheral blood at -80°C using the DNeasy Blood & Tissue kit, Quick Start Protocol (Qiagen, Chatsworth, CA, USA) according to the manufacturer's instructions. The DNA concentration and purity were checked spectrophotometrically at 260 nm and 280 nm.

### Standard curve and DNA quantification

The standard curve was constructed from the parasite DNA of a patient diagnosed with VL. The DNA of the parasites on day 5 of culture was extracted and quantified. A calculation was then made considering the size of the haploid genome of *L*. *infantum* to determine the DNA concentration corresponding to 100,000 copies of the DNA polymerase gene. From this concentration serial dilutions (10^5^, 10^4^, 10^3^, 10^2^, 10^1^, 100, and 10^−1^) were performed.

Quantification of *L*. *infantum* in peripheral blood was determined via qPCR, in which the hydrolysis probe technology (TaqMan) was used. The probe used was the one corresponding to the DNA polymerase gene, which is a single copy gene (GenBank Access AF009147) according to Bretagne and colleagues [18]. The reaction was performed using forward primers, 5'-TGTCGCTTGCAGACCAGATG-3'; and reverse primers 5'-GCATCGCAGGTGTGAGCAC-3' and probed at 5'FAM-CAGCAACAACTTCGAGCCTGGCACC-3'TAMRA. The amplification was performed in duplicate in a final volume of 20 μL containing the reagents: 2 μl of DNA from the samples, 1 μl of the probe (2.5 pmol), 1 μl of each primer (10 pmol), 10 μl of Master Mix (Applied Biosystems) and 5 μl of sterile ultrapure water. After the initial denaturation of 10 minutes at 95°C, the samples were subjected to 40 cycles of amplification consisting of two steps: 15 seconds at 95°C and 1 minute at 60°C. Negative control was included in the reaction. The reaction was run on the 7500 Fast Real-Time PCR System (Applied Biosystems).

### Whole blood stimulation assay with SLA

Samples of whole blood (500 μL) were stimulated with 10 μg/ml SLA and, incubated at 37°C for 24 hours, parallel control without stimulation was, also, incubated, after incubation time, were centrifuged at 2000 x g for 10 minutes and, the supernatant was collected and stored at -20°C for further analysis of biomarkers [16].

### Quantification of the IP-10 and MIG biomarkers

IP-10 and MIG were quantified in this study in 50 μL of plasma from whole blood SLA-stimulated or non-SLA-stimulated using the BD Cytometric Bead Array Human Flex Set (Becton Dickinson Biosciences, USA) as instructed by the manufacturer. Each sample was incubated for 1 hour at room temperature with 50 μl of capture beads and, after incubation, 50 μl of detection antibody was added and incubated for 2 hours at room temperature. The data were acquired using a FACSCanto II flow cytometer and analyzed using the Flow Cytometric Analysis Program Array (Becton Dickinson Biosciences, USA]. The results for each biomarker were expressed by the difference in plasma concentration between SLA stimulated and control in pg/mL.

The concentration of the biomarkers was compared using the Mann-Whitney U test. Significance was set at p ≤0.05. All calculations were performed using GraphPad Prism 7.0 software (GraphPad Software, USA) [16].

## Results

### Characteristics of volunteers

The volunteers in all groups were 18 years old or older. The mean age of the group of infected individuals presenting with active VL (VL) or after treatment of VL (AT) was 40 years, while for all other individuals who were living in the endemic area, before testing to separate asymptomatic infections with *L*. *infantum* (ASYMP) from uninfected endemic controls (EC) the mean age was 39 years and for non-endemic uninfected controls was 32 years (Table 1).

**Table 1:** Characteristics of volunteers participating in study.

Category of Volunteers	N	Age Mean (± SD)	Sex M/F
**VL**	25	44.3 (±15.2)	23/2
**AT**	10	40.0 (±11.7)	6/4
**EC/ASYMP**	115	39.0 (±13.1)	33/83
**NC**	17	32.0 (±11.7)	7/10

VL: Patients presenting with visceral leishmaniasis before treatment; AT: Patients presenting with visceral leishmaniasis after treatment; EC: individuals from the endemic area, with or without asymptomatic infections (ASYMP); NC—healthy controls from and non-endemic for VL; Mean ± SD: mean ± standard deviation; M/F: male/female.

### Immunochromatographic (IC) tests for identification of asymptomatic infected individuals

Two different IC rapid diagnostic tests, Kalazar Detect (InBios International Seattle, WA) and OnSite *Leishmania* IgG/IgM combo (Bio Advance Diagnósticos), both of which employ the K39 antigen, a kinesin-related protein found in isolates from cases of active VL [19], were used to detect asymptomatic people in the endemic area in Piauí state, Brazil, besides examining responses of individuals presenting with active VL. In addition, the tests were applied to individuals who had been treated for VL.

The results presented in Table 2 show that 76 (19/25) and 64% (16/25) of individuals in the active VL group presented positivity in Kalazar Detect and OnSite *Leishmania* IgG tests, respectively; however, the OnSite *Leishmania* IgM test was not positive in any of these individuals. In treated individuals (AT group), some of them years after cure, reactivity was slightly lower: 50 (5/10) and 57% (4/7) were positive in the Kalazar Detect and OnSite *Leishmania* IgG tests, respectively, and none were positive in the OnSite *Leishmania* IgM test. Among those individuals who were living exposed to *L*. *infantum* (Asymp and EC groups), only 1.7% (2/115) were positive in both tests, while none of the samples in the EC group were positive with the OnSite IgM test.

**Table 2:** Immunochromatographic tests for detection of infection with *L*. *infantum*.

Tests	VL N = 25	AT N = 10	EC N = 115
	% of Positive tests		
**Kalazar Detect**	76	50	1.7
**OnSite IgM**	0	0	0
**OnSite IgG**	64	57	1.7

VL: Patients presenting with visceral leishmaniasis before treatment; AT: Patients presenting with visceral leishmaniasis after treatment; EC: individuals from the endemic area, with or without asymptomatic infections

### ELISA test for identification of asymptomatic infected individuals

**Fig 1:**
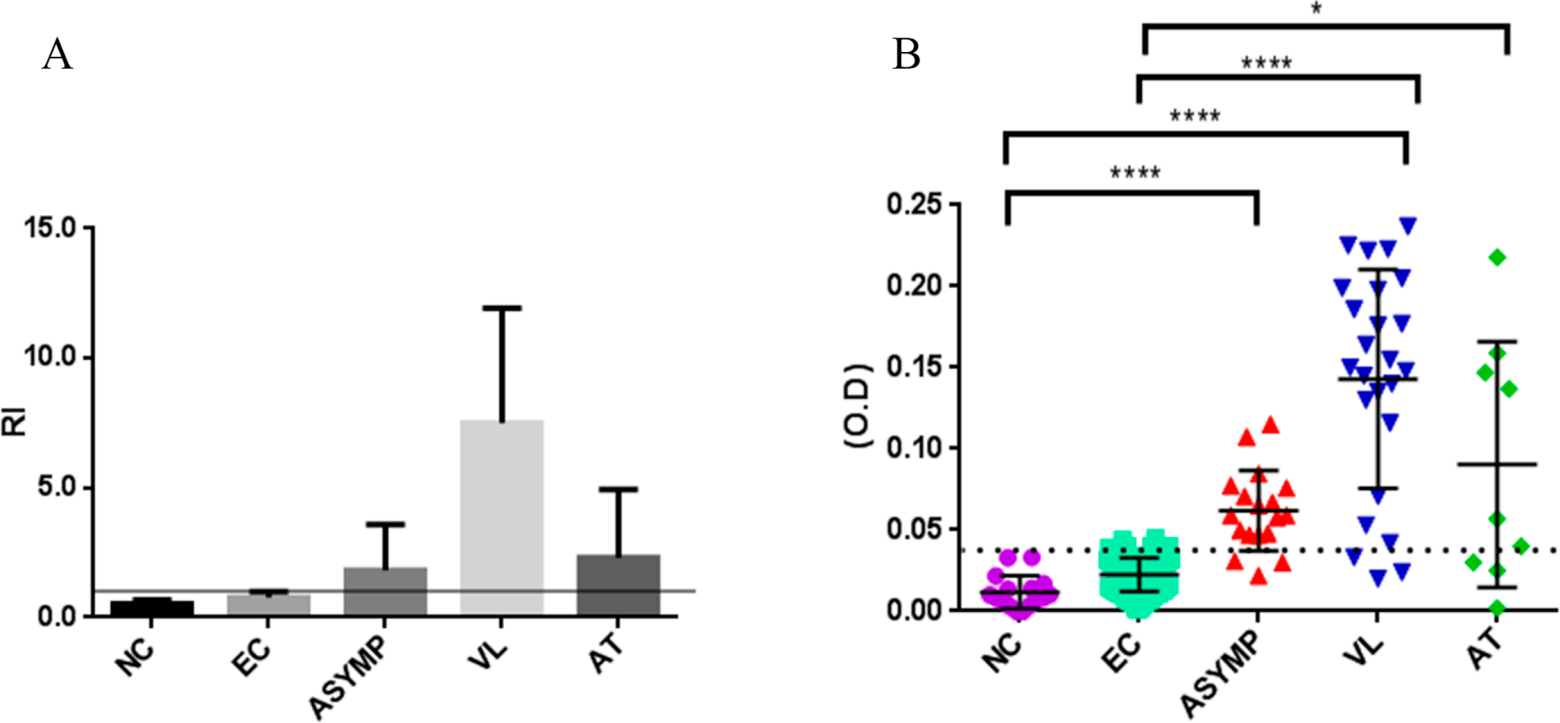
Reactivity indices (RI) and levels of soluble *Leishmania* antigen (SLA)-specific antibodies in ELISA of serum IgG at a 1:200 dilution of serum for detection of *L*. *infantum*-infected asymptomatic individuals. Fig 1A: Individuals from the endemic area were separated into the following groups according to their RI in ELISA, as described in Marques et al. 2017 [11]: non-infected, endemic control group (EC), characterized by a RI <1.1; asymptomatic infected group (Asymp), active VL group (VL) and individuals who had been treated for VL and were cured (after treatment group, AT), all with a RI ≥1.1. by RI was calculated as follows: mean of optical density each sample divided by the cutoff point of 0.04. Levels of SLA-specific antibodies from healthy individuals from a non-endemic area (normal controls, NC) and EC, Asymp, VL and AT groups as optical density (O.D.) measured at a wavelength of 450 nm. Kruskal-Wallis test followed by Dunn's ad hoc test [***, *P* < 0.0001] (B).

When ELISA technique was used with sera diluted at 1:200 and reacted with soluble *Leishmania* antigen (SLA), 84% (21/25) of seropositivity was found in the active VL group, whilst 55.5% (5/9) of the treated individuals (AT group) were seropositive. Among those individuals who were living exposed to *L*. *infantum*, 13% (15/115) were positive and may be infected with *L*. *infantum*, without, however, presenting with manifest symptoms. Provided these individuals presented a reactivity index (RI) ≥1.1, as described by Marques and colleagues [11], in which the mean of the optical density each sample is divided by the cutoff point, which was 0.04 in this study (Fig 1A), they are categorized as infected. Once all individuals infected with *L*. *infantum* were identified, symptomatic infections were then categorized based on the statistical difference between the levels of their different markers and those of the EC or NC groups. Thus, it is possible to differentiate asymptomatic infections (Asymp group), showing RI ≥1.1 from active VL and AT groups. Individuals from Teresina presenting an RI <1.1 in ELISA were considered non-infected and were categorized as the endemic control (EC) group.

As illustrated in Fig 1B, where data are plotted as optical densities (O.D) measured at a wavelength of 450 nm, levels of SLA-specific antibodies differed significantly between healthy individuals from a non-endemic area (normal controls, NC) and infected, asymptomatic individuals from the endemic area (Asymp) (Kruskal-Wallis test, p-value < 0.0001), and between the NC group and patients with VL, whether active VL (VL) or treated VL (AT) (Kruskal-Wallis test, p-value <0.05). Furthermore, individuals with disease, whether active or after treatment, presented significantly higher levels of SLA-specific antibodies than endemic controls (EC), but not when compared with levels of SLA-specific antibodies seen in the Asymp group (Kruskal-Wallis test, p-value < 0.0001).

### qPCR for identification of parasite DNA in asymptomatic patients

The qPCR technique was performed to detect the presence of *L*. *infantum* in the peripheral blood from patients with active VL and other groups from the endemic area confirming, in this manner, asymptomatic individuals.

**Fig 2:**
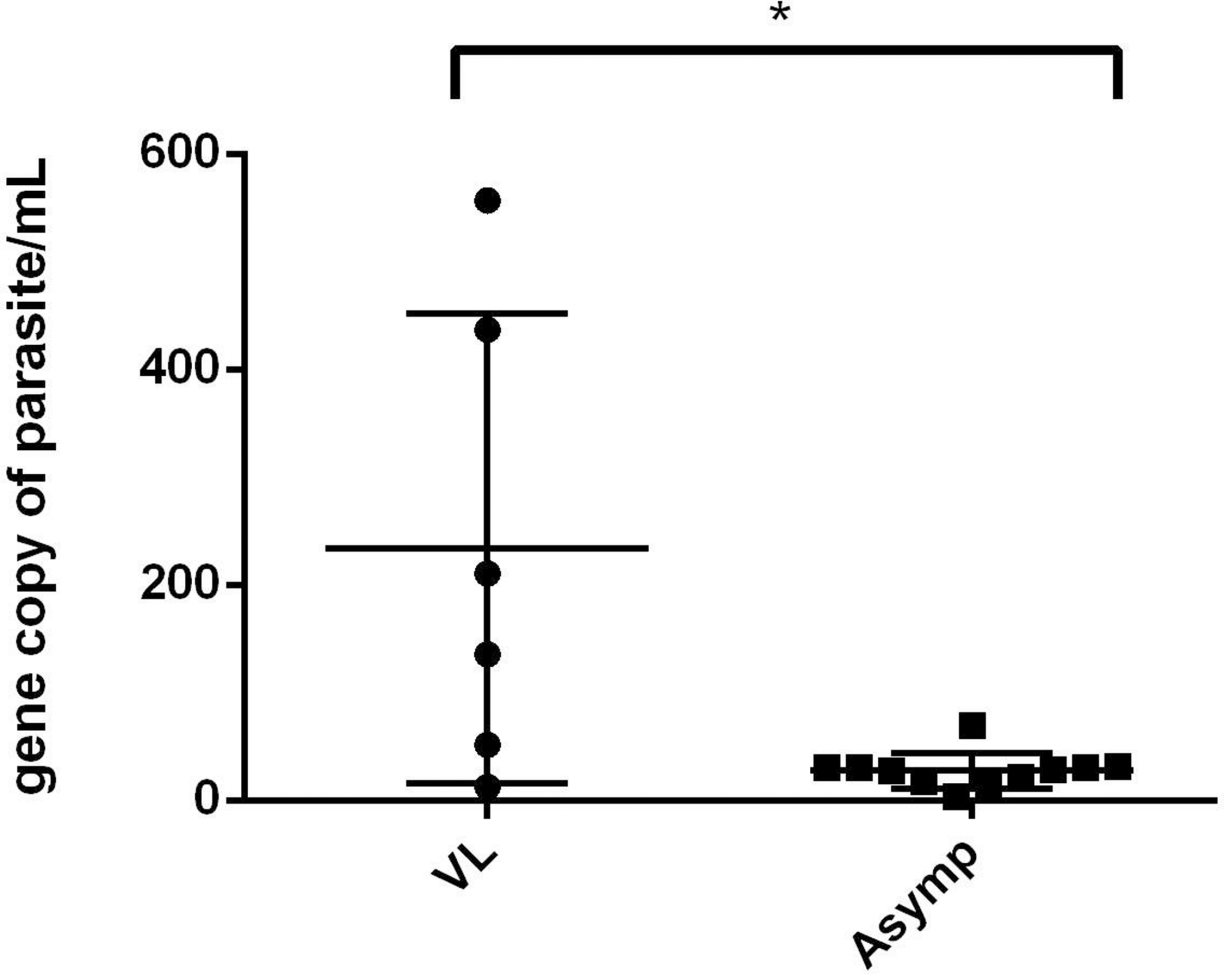
Parasite loads of patients with active visceral leishmaniasis and asymptomatic infected individuals. The gene copies of parasites/mL were compared between patients presenting active visceral leishmaniasis (VL) and individuals with asymptomatic infections of *L*. *infantum* (Asymp). Mann Whitney U test P = 0.0273.

We selected at random 29 individuals living in the endemic area among 115 volunteer participants in this study and 11 (37.9%) of those were classified as being latently infected with *L*. *infantum* by means of the presence of gene copies of parasites/mL on qPCR. Additionally, 6 (60%) of the 10 individuals with active VL analyzed were positive. The results depicted in Fig 2 show that the number of gene copies of *Leishmania* differed significantly (Mann Whitney test, p-value < 0.05) between the Asymp and active VL groups. The slope of the reaction was -3.33 and the efficiency was 99.5%; the negative reactions were not represented.

### Quantification of the biomarkers IP-10 and MIG in *L*. *infantum*-specific lymphocyte responses of infected asymptomatic individuals

For further analysis of individuals infected with *L*. *infantum* in the population from the endemic area, the concentrations of the IP-10 or MIG biomarkers were determined after stimulation with SLA of peripheral blood obtained from 14 patients with active VL, 6 cured individuals and 30 from the 115 healthy individuals who were living exposed to *L*. *infantum* without developing any symptoms of the disease; unstimulated blood was used as control for analysis. In addition, we also sought to validate in a Brazilian population the results from a study by Ibarra-Meneses and colleagues that described these cytokines as biomarkers of infections with *L*. *infantum* and *L*. *donovani* in individuals from Spain and Bangladesh, respectively [16].

**Fig 3:**
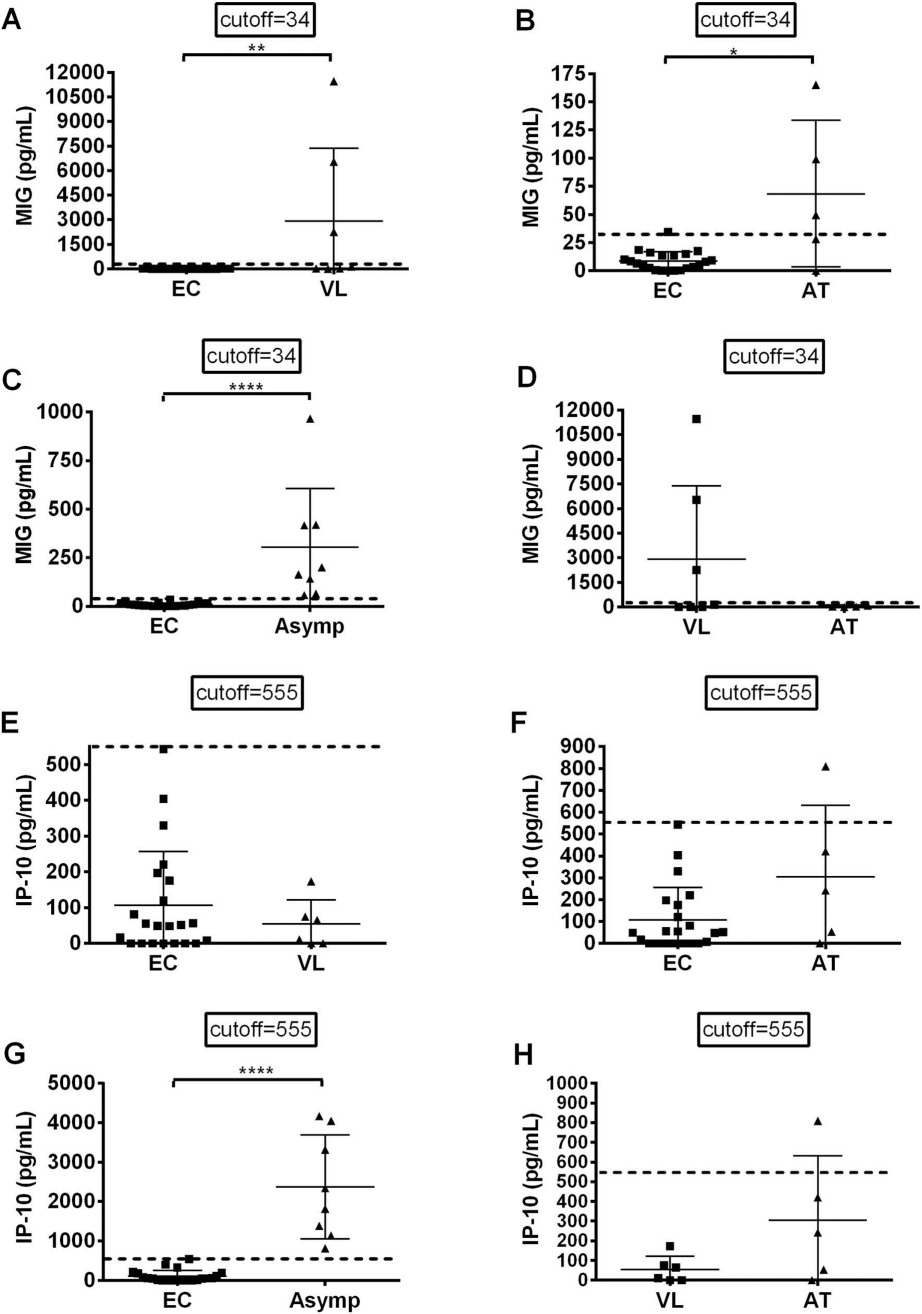
Levels of MIG and IP-10 biomarkers in SLA-stimulated cultures of whole blood of individuals presenting with active Visceral Leishmaniasis (VL), after treatment Visceral Leishmaniasis (AT), with asymptomatic infections (ASYMP) and healthy people from the endemic area (EC) of Teresina, Brazil. The MIG concentrations (A-D) and IP-10 concentrations (E-H) are pg/mL. Each individual is represented by one dot; means and cutoffs (mean plus 3 times standard deviation) are represented by horizontal and dotted lines, respectively. The non-parametric Mann-Whitney U test was used to compare differences between the groups. **** *P* < 0.0001; ** *P* < 0.05; * *P* < 0.05.

The results presented in Fig 3 show that by using both biomarkers it was possible to detect 8 (26.7%) responsive asymptomatic individuals among 30 individuals analyzed from the endemic area; these individuals were also categorized in the Asymp group. Significant differences in quantities of MIG (Fig 3C) (t test, p-value <0.0001) or IP-10 (Fig 3G) (t test, p-value<0.0001) were observed between non-infected, healthy endemic controls (EC) and asymptomatic infected individuals (Asymp). The concentrations of MIG in active VL and AT groups differed significantly (t-tet, p = 0.0093 [Fig 3A] and p = 0.0337 [Fig 3B], respectively) when compared to those of endemic controls (EC). No significant difference was found in active VL versus AT group (Fig 3D). Concentrations of IP-10 in reactions of cells from the active VL group (Fig 3E) and AT group (Fig 3F) did not differ significantly when compared to the reactions of cells from the EC group, the same occurring between active VL and ATgroups (Fig 3H).

## Discussion

Teresina, in Piauí state, is one of the urban areas in Brazil where the population is at risk for developing VL [20], however Public Health authorities lack a diagnostic routine to identify asymptomatic individuals infected with *L*. *infantum*. Such a routine would be important in such a context for following HIV-positive individuals, to assess transmission force and herd immunity and to evaluate false positives for other diseases. In this study, we evaluated different methods to detect asymptomatic individuals in that endemic region. We found that the IC tests employed in this study are not efficient for identifying this group of individuals because only 1.7% of the healthy participants were positive. However, these tests remain as an important alternative for debilitated patients who cannot undergo bone marrow aspirate procedures: in this study positive results were found to be above 60% and 70% for the two tests in cases of active VL. We obtained satisfactory results when qPCR was used for detection of asymptomatic individuals. ELISA, in turn, determined that 13% of individuals in the healthy population living exposed to *L*. *infantum* were infected and was the test that best concurred with results of qPCR. Finally, the biomarkers IP-10 and MIG indicated that 26.7% of individuals from an endemic area were infected, being that 62.5% of these (5 of 8) concurred with ELISA and qPCR tests.

IC tests used for serodiagnosis of active VL are based on rK39, a recombinant protein derived from a specific antigen produced by *Leishmania donovani* complex [21]. Several studies have evaluated the efficacy of rapid tests IC for diagnosis of active VL with regard to performance, cost, acceptability and suitability [22] for both of the agents that cause VL. In this study, we report that Kalazar Detect and OnSite *Leishmania* IgG/IgM were able to detect 76% and 64% of patients with clinically active VL caused by *L*. *infantum*; this performance is lower as compared with other studies, which reported 91.2% sensitivity and 94.5% specificity when using OnSite *Leishmania* IgG/IgM [23], and 88.1–100% sensitivity and 90.6–99.1% specificity when using Kalazar Detect [24–26]. It has already been reported that the performance of similar diagnostic products may differ according to geographical regions due to the fact that *L*. *infantum* might be less abundant in blood than *L*. *donovani* [27], due to parasite antigenic diversity and/or differences in antibody concentrations. These latter differences, in turn, may be due to different age patterns, immune responses, and nutritional status of patients [28]. Indeed, the study by [23] employed a historical collection of sera from Brazilian individuals to evaluate these IC tests. These authors did not inform the periods that the sera cover, but it is safe to say that the important and relatively recent epidemiological transition that Brazil has undergone changed its population’s nutritional status, access to sanitation and many other aspects that affect performance of antibody-based responses, including performances of antibody-based diagnostic tests. Furthermore, we refer readers to the important recent study by Kityo and colleagues that elucidated the mechanism whereby different populations mount distinct responses to identical antigens according to their levels of immune activation [29]. In addition, median ages of the cohorts differ between the studies that compare these tests.

This study employed OnSite *Leishmania* IgG/IgM and Kalazar Detect because they fulfilled the Brazilian government’s public call for purchase of the new rapid tests for VL in that country, which stipulated the minimum performance as 90% for sensitivity and specificity [23,30]. The fact is that in Brazil reproducibility of all IC tests for diagnosis of VL is low and these tests need further evaluation [31].

In addition to issues of performance of tests in active and treated VL in patients from different geographical areas, there is the issue of their performance in asymptomatic individuals infected with *L*. *infantum*. Active VL, but not asymptomatic infections, are accompanied by inflammation [32,33] and polyclonal activation of B cells [34], responses which may facilitate targeting the k39 antigen, thus explaining why the tests based on this antigen do not detect asymptomatic infections. Furthermore, *L*. *infantum* remains in the blood years after it is first detected, which may explain the positive results of the IC test in the AT group [27,35]. On the other hand, asymptomatic individuals have been infected for an unknown period during which *Leishmania* may circulate in the peripheral blood episodically, detectable by PCR, but not necessarily inducing a humoral or cellular response [35–39]. Indeed, Kalazar Detect cannot detect asymptomatic individuals infected with *L*. *infantum*, but it is able to detect infected asymptomatic individuals in regions where *L*. *donovani* is presented [16,40], as in a hyper-endemic area from Bangladesh, albeit with low sensibility: Kalazar Detect detected only one positive serum sample among 35 individuals exposed to the parasite. On the other hand, the OnSite test was able detect asymptomatic with a slightly higher sensitivity, but only in fresh serum samples and not in stored serum [41].

In Brazil, asymptomatic infection was detected in the same region of this study by PCR-based assays of blood [10]. qPCR is a complex technique that is not standardized, although it is reported to present high sensitivity and specificity, both in blood and bone marrow samples [27]. Furthermore, it is too expensive for the routine application in an endemic area due to high cost of reagents and equipment, however the method has progressed in recent years. This would require greater accessibility of reagents, miniaturization of equipment, etc., so that routine use of the technique is possible in less favored areas [42]. A promising possibility for this to be achieved is the qPCR performed with blood dried on cellulose paper [43,44]. The qPCR can be an important tool to identify asymptomatic infections in endemic areas, because it is a highly sensitive test to detect significant parasitemia, to recognize of potential progressers to clinical disease, facilitate early intervention, avoid possibility of disease transmission and for genotyping of species of interest in a given geographical area [41,45–49]. Thus, despite the fact that PCR may be of less value as a marker for acute clinical disease in VL endemic areas, it is a good marker of infection [27].

Antibody-based detection of infections with *L*. *infantum* in endemic areas also faces limitations, but can also identify healthy infected individuals [27]. However, serological tests such as direct agglutination (DAT) could be useful in the field [50,51] and, with efforts to improve technician skills and laboratory facilities, it could become a perfect tool for detection of the asymptomatic population. DAT reativity is controlled mainly by the host’s cell-mediated immune response, which highlights the importance of researching new markers [27]. Automated ELISA tests promise to be very efficient [52], but ELISA is considered to be a diagnostic tool of low sensitivity for detection of asymptomatic human infections with *L*. *infantum* and false-negative test results underestimate the actual rate. However, ELISA can identify asymptomatic infections as is shown in the present study and also in other studies [6,53,54]. In addition, the ELISA method using crude antigen is generally more sensitive, albeit less specific [11]. Our results show a great coincidence of positivity of ELISA when compared with qPCR and IP-10/MIG biomarkers tests for the detection of asymptomatic individuals. Since ELISA is based on a multicomponent antigen, SLA, this fact may explain why it is a more sensitive test for detecting asymptomatic individuals than single antigen-based IC tests.

In this study the concentrations of the MIG biomarker were significantly higher in supernatants of blood cells responding to SLA from individuals in active VL, AT and Asymp groups, compared to those in the EC group and was higher in VL patients, followed by asymptomatic infections and lower in AT group; no significant differences were observed between active VL and AT groups. On the other hand, concentrations of the IP-10 biomarker, interestingly, were higher in supernatants of blood cells responding to SLA from individuals presenting with asymptomatic infections compared to those seen in samples from the active VL group and the AT group. Thus, chemokines MIG and, especially, IP-10 can indeed provide a sensitive means of detecting a specific T-cell response to antigen following *Leishmania* infections in Brazil, in the same manner as has been shown in studies conducted in regions endemic for *L*. *infantum* in Spain and for *L*. *donovani* in Bangladesh [16,40]. IP-10 is released in response to both type I and type II IFN and is a chemotactic factor for activated T cells and NK cells, which when thus stimulated subsequently express CXCR3 and are recruited to sites of tissue inflammation [55]. This is one mechanism of resistance to *L*. *infantum* mediated by IP-10; in addition, this cytokine also mediates protection against *Leishmania* by inducing nitric oxide, an important leishmanicidal mediator [56–59]. These mechanisms mediated by IP-10 may explain the higher levels of this cytokine seen in the ASYMP group, which is seems to be resistant to developing disease despite infection. These facts about this biomarker raise questions concerning the mechanisms that result in resistance to development of disease, seen in the majority of infections of humans with *L*. *infantum*. The cultures of stimulated cells are performed with circulating SLA antigen-reactive leukocytes. Transcriptomes of whole blood leukocytes have been useful not only for identifying disease-specific biomarkers [60,61], but also for unraveling mechanisms of resistance and susceptibility to developing disease after infections with *L*. *infantum* [62]. In this study, IP-10 seems to indicate that the immune response is well established regarding recruitment of activated/effector T cells, thereby initiating the effector arm of Th-1 cell immunity and differentiating active VL that asymptomatic infection. In conclusion, IP-10 showed high sensitivity and specificity to identify asymptomatics in *L*. *donovani* and *L*. *infantum* areas, meaning it is a good biomarker [16].

Since there is no standard diagnostic for the detection of asymptomatic infection, the combination of different techniques to determine their real prevalence is currently the best option [16,63]. rK39 IC, direct agglutination and leishmanin skin tests detected, respectively, 1.5%, 5.3% and 5.6% of asymptomatic infection in study performed in selected villages from Libo Kemkem and Fogera districts (Amhara State, Ethiopia), indeed 10.1% of asymptomatic infection was detected with combined use of serologic methods and leishmanin skin test [63]. It is important to also use DNA detection tests, since asymptomatic infected individuals are carriers the *Leishmania* infection [27].

We must point out that a previous study performed with different samples from the same the same population examined in the present study [53] found a significantly higher prevalence of asymptomatic individuals with positive cellular skin reactions to Montenegro antigen than the number found with the *in vitro* cellular reaction employed in the present study (60.41% *vs* 26.7%, respectively; chi-square statistic 5.9997; *p*-value 0.0143). While this difference may reflect the transmission dynamics of two periods separated by six years, there are also important differences between the two cellular tests: the Montenegro reaction results from the global cellular response elicited by the antigen, while the *in vitro* cellular reaction accounts for the production of two cytokines in this study and four cytokines in the study by Ibarra-Meneses and colleagues [16]. In our previous study we also showed that, of nine cytokines evaluated, only the levels of TNF-α differed significantly between individuals with asymptomatic infections and endemic healthy, non-infected controls [53]. In view of these results we suggest that levels of TNF-α also be evaluated in futures studies employing the *in vitro* cellular test.

In conclusion, the present study shows that qPCR can be an efficient tool to distinguish asymptomatic individuals infected with *L*. *infantum* among healthy individuals living in Teresina, Piauí, Brazil, an endemic area for VL. In addition, ELISA to detect *L*. *infantum*-reactive antibodies continues to be a standard method to use in the field. The biomarkers MIG and IP-10 showed equivalent results compared to the qPCR and ELISA, with highlights for IP-10 for indicating asymptomatic infections with *L*. *infantum*. In summary, healthy individuals living in Teresina, Brazil and exposed to *L*. *infantum*, were evaluated for subclinical infection with serological, molecular and cellular approaches: among 115 of these individuals, 15 (13%) presented antibodies reacting with SLA in an ELISA; gene copies of parasites detected with qPCR were present in 11 (37.9%) of 29 of these individuals; finally, leukocytes from 8 (26.7%) of 30 of these individuals when treated with SLA responded by producing significant amounts of IP-10 and MIG when compared with their untreated cells.

Healthy, asymptomatic individuals from endemic areas of VL were the focus of this study because they represent the majority of outcomes of infections with *L*. *infantum* and for this reason they can help elucidate the immune mechanisms that operate in this outcome. We are not proposing to use any of the tests employed in this study for routine evaluations in the clinic (e.g., for blood banks). However, we need to identify these individuals in the endemic area for the reasons stated above. This is not an easy task without employing the current alternatives. Understanding this group, may however lead to development of tests and biomarkers applicable in the clinic. So far, few studies have been focused on individuals bearing latent infections with *L*. *infantum* and the results presented here justify further investments to definitely validate biomarkers for this group.
